# The effect of i-deals on employees’ unethical behavior during the COVID-19 pandemic: The roles of hubristic pride and grandiose narcissism

**DOI:** 10.3389/fpsyg.2022.938864

**Published:** 2022-09-01

**Authors:** Zhihao Liu, Xiaoyan Zhang, Hanzhi Xu, Hui Deng, Jiajia Li, Yuanyuan Lan

**Affiliations:** ^1^School of Business, Qingdao University, Qingdao, China; ^2^School of Economics and Management, Beijing Jiaotong University, Beijing, China; ^3^School of Music and Recording Arts, Communication University of China, Beijing, China; ^4^School of Fine Arts, Qingdao University, Qingdao, China; ^5^College of Design and Innovation, Tongji University, Shanghai, China

**Keywords:** COVID-19 pandemic, idiosyncratic deals, hubristic pride, unethical behavior, grandiose narcissism, social cognitive theory

## Abstract

The COVID-19 pandemic has created enormous challenges for organizations and employees. Due to the effectiveness of idiosyncratic deals (i-deals for short) in management practices, more and more organizations use this human resource management tool to address the challenges posed by the COVID-19 pandemic. However, whether there are potential risks or negative effects of i-deals in the COVID-19 pandemic environment is not very clear. Drawing upon social cognitive theory, we proposed that i-deals may foment focal employees’ unethical behavior by triggering their hubristic pride, and such process may be moderated by their trait of grandiose narcissism. We conducted a survey during the COVID-19 outbreak and tested our hypotheses with 492 samples from Shandong Province, China. Consistent with predictions, we found a positive relationship between i-deals and hubristic pride, which, in turn, increased their unethical behavior. And the relationship between i-deals and unethical behavior was mediated by hubristic pride. Furthermore, grandiose narcissism strengthened the positive relationship between i-deals and hubristic pride, as well as the indirect effect of i-deals on unethical behavior via hubristic pride. Our findings contributed to the literature on i-deals and provided guidance for organizations to address the challenges posed by the COVID-19 pandemic.

## Introduction

COVID-19 is not only a global health crisis but also a huge threat to the management of organizations. Current studies have shown that the ongoing COVID-19 pandemic affected employees’ occupational health and safety in many respects (Rudolph et al., 2021), including increasing job insecurity (Latorre et al., 2021), lowering work engagement and accountability (Liu et al., 2021), and causing some mental health problems such as anxiety (Trougakos et al., 2020), thus seriously impacting the performance of organizations. These unprecedented challenges have forced managers to rethink current management strategies and seek out solutions to meet the changing and unpredictable needs of key employees (Obenauer, 2021). I-deals, the voluntary and non-standardized employment agreements that are negotiated by individual employees with their employers (Rousseau et al., 2006), can flexibly meet the various needs of employees. For example, i-deals recipients have access to flexible working time or locations, training and promotion opportunities, and therefore have been seen as an appropriate tool to address some issues caused by the COVID-19 pandemic (Obenauer, 2021). During the outbreak of COVID-19, the use of i-deals in organizations has increased significantly. A Gartner (2020) survey pointed out that half of the surveyed organizations reported that more than 80% of their employees are working from home after the COVID-19 pandemic outbreak, whereas only 30% of employees worked from home before the pandemic. Another report from Pricewaterhouse Coopers [PwC] (2020) showed that many organizations have provided employees with personalized development opportunities and career planning to attract top talent. These measures can help organizations survive the COVID-19 pandemic and seize the opportunity to catch up with competitors to some extent.

Empirical research has found that i-deals have positive impacts on focal employees. To begin with, i-deals have been manifested to benefit recipients’ work attitudes. For example, i-deals can enhance focal employees’ vigor, gratitude (Ng et al., 2021), organizational commitment and job satisfaction (Liao et al., 2017), and reduce their cynicism (Ng et al., 2021) and turnover intention (Ho and Tekleab, 2016; Ng, 2017; Zhang et al., 2021). In addition, considerable studies have shown the benefits of i-deals in terms of work-related behaviors. For example, empirical research indicated that i-deals could promote focal employees’ voice behavior (Ng and Feldman, 2015), helping behavior (Guerrero and Challiol-Jeanblanc, 2016), and organizational citizenship behavior (Anand et al., 2010). Specifically, research by Ng and Feldman (2015) revealed that when managers and professionals from both the United States and China received i-deals, they were motivated to conduct more voice behavior. Moreover, recent research also examined the positive effects of i-deals in the context of the COVID-19 pandemic. For example, Tsukamoto (2021) found that location flexibility i-deals could lead to a great degree of self-determination and higher productivity. And Latorre et al. (2021) contended that flexibility i-deals could improve sustainable well-being at work and performance during the Brazilian COVID-19 pandemic.

Although there are numerous studies investigating the positive effects of i-deals, we still know very little about their negative effects, with only a little research shedding light on this issue. For example, current studies indicated that i-deals might cause coworkers’ feelings of unfairness (Rousseau et al., 2006), envy or emotional exhaustion (Ng, 2017; Kong et al., 2020), complaining behavior (Marescaux et al., 2019b), and perception of status threats (Zhang et al., 2020). Nevertheless, thus far, the most of proven negative effects are recognized from the coworkers’ perspective, and the exploration of potential negative effects of i-deals on the receivers is still in its infancy. In particular, as the outbreak of the COVID-19 pandemic creates a challenging external environment for employees and organizations (Hamouche, 2021), the application of i-deals in organizations has increased significantly (Gartner, 2020; Latorre et al., 2021). In such a stressful context, the effects of i-deals in organizations may be more complex (Latorre et al., 2021). Furthermore, although related research on i-deals has mainly focused on social exchange theory (Anand et al., 2010; Ng and Feldman, 2015), Liao et al. (2016) pointed out that social exchange theory is limited in explaining the impact of i-deals. Especially in the context of the COVID-19 pandemic, i-deals recipients’ work environment changes a lot. Whether and how this change in the environment affects employees’ cognition and behavior is unknown. To sum up, in order to enrich our understanding of the fuller effects of i-deals, a deeper investigation is warranted.

In this study, we develop a moderated mediation model based on social cognitive theory to explore the mechanisms through which i-deals might influence the receivers’ cognition and subsequent behavior in the context of the COVID-19 pandemic. Social cognitive theory suggests that the relationships between the external environment, individuals’ subjective cognition, and behavioral outcomes are determined interactively (Bandura, 1986). According to this core assumption, the successful negotiation of i-deals, as a change in the external work environment, may change individuals’ subjective cognition. Specifically, i-deals are characterized by heterogeneous and scarcity that have many potential implications. For example, being able to negotiate i-deals with supervisors is a sign of an employee’s valuable, contribution, potential, or acceptance (Rousseau et al., 2006). Especially, as the COVID-19 pandemic has caused a large number of layoffs and increased unemployment (Eurostat, 2020), it will be scarcer to negotiate i-deals with leaders. Therefore, employees who receive i-deals in the context of the COVID-19 pandemic may believe that they obtain i-deals due to their own abilities, which will lead to more hubristic pride (Tracy and Robins, 2007). In addition, social cognitive theory posits that individuals’ cognition of external events shapes their subsequent behaviors. Given that individuals with hubristic pride often display anti-social attitudes and misbehavior (Tracy et al., 2010), we further propose that hubristic pride may result in unethical behavior.

**FIGURE 1:**
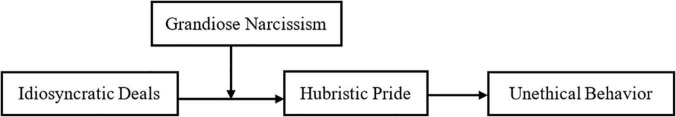
Conceptual model.

Social cognitive theory also points out that individuals’ cognition and behavior are not only affected by the external environment, but also differ due to their characteristics (Bandura, 1986). Previous research has shown that the understanding of the acquisition of i-deals may vary among individuals (Liu et al., 2013). Therefore, we speculate that the relationship between i-deals and unethical behavior is affected by individual trait differences. Studies have found that grandiose narcissistic individuals tend to overestimate their own abilities (Wink, 1991; Miller et al., 2011), which may affect individuals’ perception of the external environment and hubristic pride (Tracy and Robins, 2007). Therefore, we believe that higher grandiose narcissistic employees are likely to experience more hubristic pride after obtaining i-deals. Taken together, drawing on social cognitive theory we attempt to investigate the relationship between i-deals and recipient employees’ unethical behavior by uncovering the potential cognitive mechanism of hubristic pride and the moderating effect of grandiose narcissism in the context of the COVID-19 pandemic. The theoretical model is shown in Figure 1.

Our study advanced existing research in three specific ways. First, we enriched the understanding of i-deals negotiated in the context of the COVID-19 pandemic by investigating the negative impacts of i-deals on the receivers. While most of the past research has demonstrated i-deals’ positive effects, we have no idea about whether i-deals may produce potential negative effects. Considering that the effects of i-deals negotiated during the COVID-19 pandemic will be more complex (Latorre et al., 2021), we explore whether and how i-deals impact i-dealers’ unethical behavior in this context. Second, we clarify the mechanism through which i-deals may trigger recipient employees’ unethical behavior and the boundary condition that may constrain this effect, thus enriching the literature research on i-deals. Research on the COVID-19 pandemic has indicated that the COVID-19 pandemic could negatively affect individuals’ emotions and psychological states (Min et al., 2021). By exploring the mediating role of hubristic pride and the moderating role of grandiose narcissism, this research enhances our knowledge of how and when i-deals may lead to negative effects. Finally, we enrich the i-deals literature by applying social cognitive theory. Specifically, from the perspective of social cognition, we explore the downstream effects of i-deals from the “environment-cognitive-behavior” path, which provides a new perspective on understanding the effects of i-deals.

## Literature review and hypotheses

### I-deals, hubristic pride, and unethical behavior

As an unprecedented health crisis, the COVID-19 pandemic has severely impacted organizations and employees, throwing them into great fear and uncertainty (Hamouche, 2021). In this context, more i-deals have been negotiated between organizations and employees (Gartner, 2020), wishing to sustain the smooth functioning of organizations and improve employee performance and loyalty (Ho and Kong, 2015). I-deals are special employment terms negotiated by individual employees with employers that can meet both of their needs (Rousseau et al., 2006). It has been confirmed that the overall degree of i-deals in the team positively affects team performance (Anand et al., 2022). However, by their very nature, i-deals are individually negotiated, and their purpose has always been to attract and retain top talent, which implies that not all employees have access to i-deals (Rousseau et al., 2006). This makes i-deals characterized by scarcity and importance (Xia et al., 2021), especially in the complex external environment of the COVID-19 pandemic.

When employees themselves are credited as the cause of a successful event, they will inspire a sense of pride. Unlike other basic human emotions, such as happiness, sadness, or anger, pride often means less reflection of one’s true feelings and self-assessment, such as self-exaggeration (Yeung and Shen, 2019). Tracy and Robins (2004) pointed out that pride is triggered by individual cognitive processes. And pride is a broad concept that composes of two distinct emotions, namely authentic pride and hubristic pride (Tracy and Robins, 2007). People with authentic pride believe that advantage comes from intrinsic, unstable, and controllable efforts, while people with hubristic pride believe that advantage comes from intrinsic, stable, and uncontrollable ability. Since hubristic pride is an emotion based on beliefs about one’s own abilities (Tracy and Robins, 2004, 2007), it is easily triggered by an individual’s cognitions that abilities lead to i-deals. Previous research shows that hubristic pride can be stimulated by childhood maltreatment (Li and Xiang, 2020), which in turn is associated with more abusive behaviors (Yeung and Shen, 2019) and antisocial behavior (Stanger et al., 2021).

One key tenet of social cognitive theory is that individuals’ cognitions could be determined by environmental impacts (Bandura, 1983, 1989, 1990). Given that i-deals is an important environmental factor (Zhang et al., 2021), following social cognitive theory, we speculate that employees receiving i-deals may trigger their hubristic pride. Specifically, on the one hand, the purpose of leaders negotiating i-deals with their employees is to recruit, motivate and retain valuable employees (Rousseau et al., 2006). In the workplace, only a few employees (i.e., highly skilled professionals, key position employees, or high-performance employees) can successfully negotiate i-deals with leaders (Rousseau et al., 2006). Such truth makes employees who receive i-deals believe that they are talented and capable. Thus, they are likely to experience hubristic pride. On the other hand, the successful negotiation of i-deals implies that focal employees can enjoy more competitive and limited organizational resources than others, which endows i-deals with many hidden meanings (Greenberg et al., 2004). Specifically, i-deals recipients may have higher organizational status, more trust, and more attention from leaders (Rousseau et al., 2006; Ng, 2017). These cues can enhance their assessment of their own abilities (Zhang et al., 2021), and then stimulate their hubristic pride. Thus, we posit that:

H1: I-deals are positively related to hubristic pride.

Unethical behavior refers to the organizational members’ action that has a harmful effect on others, which is generally illegal or morally unacceptable (Jones, 1991), such as theft, sabotage, lying to customers, and misrepresentation in financial reports. Kish-Gephart et al. (2010) pointed out that some negative workplace behaviors, such as being late, are not included, as they do not violate the widely accepted ethics. However, some studies suggest that time theft, such as wasting or not performing work during scheduled work hours, is also an unethical practice (Henle et al., 2010; Paterson and Huang, 2019). Such behavior is unethical since employees steal work time that belongs to the organization and do not work for the organization during this time (Henle et al., 2010). Employees’ unethical behavior exists widely in various social organizations, such as enterprises, governments, and academic organizations (Peterson, 2004). It can cause immeasurable harm to the organizations’ long-term performance and sustainable development (Treviño et al., 2006).

According to social cognitive theory, individuals’ thoughts, beliefs, and feelings could shape their behavior (Bandura, 1986). We, therefore, propose that increased hubristic pride may elicit employees’ unethical behavior in the context of the COVID-19 pandemic. Specifically, it has been proven that hubristic pride may be associated with negative personalities and behaviors (Tracy et al., 2010). First, employees with high hubristic pride are more likely to be angry and hostile toward others (Tracy and Robins, 2004; Carver et al., 2010), and have lower levels of conscientiousness (Cheng et al., 2010). Thus, when employees experience high hubristic pride, they are prone to conduct unethical behavior. Second, studies have shown that employees with hubristic pride are aggressive (Tracy et al., 2010). Those employees have great prejudice and discrimination against the outside world (Ashton-James and Tracy, 2012), and have a strong sense of control toward others (Baumeister et al., 2000). Thus, it is reasonable to predict that hubristic pride may lead to unethical behavior. Furthermore, hubristic pride has been found to positively predict antisocial behavior (Krettenauer and Casey, 2015). For example, individuals with hubristic pride are likely to engage in behaviors such as cheating and fraud in order to increase their chances of achieving their goals (Magnan et al., 2008; Bureau et al., 2013). Given that hubristic pride could reduce individuals’ moral judgment and prosocial motivation (Verbeke et al., 2004; Kim and Johnson, 2014), we speculate that hubristic pride may increase employee unethical behavior. Therefore, we propose the following hypothesis:

H2. Hubristic pride is positively related to unethical behavior.

Social cognitive theory points out that individuals acquire information from the external environment and construct their cognitions about the information, and individual behavioral decisions are the result of the synergy of individual cognitions and environmental factors (Bandura, 1989). “Cognitive regulation” is the mediating mechanism that transmits the influence of external environmental factors on individual behaviors (Bandura, 1986, 1991). Therefore, based on social cognitive theory and hypotheses 1–2, we propose the mediating role of hubristic pride in the relationship between i-deals and unethical behavior. Specifically, employees will experience a series of psychological and cognitive changes after receiving i-deals, such as a belief that they are superior (Rousseau et al., 2006). Such progress will arouse their hubristic pride. Since hubristic pride is usually associated with anti-social behaviors such as fraud and theft (Magnan et al., 2008; Bureau et al., 2013), individuals who experience hubristic pride are expected to participate in more unethical behavior. Therefore, we propose the following hypothesis:

H3: Hubristic pride mediates the relationship between i-deals and employees’ unethical behavior.

### The moderating role of grandiose narcissism

In the past few decades, narcissism has received increasing attention as a sub-clinical individual difference (Ames et al., 2006). Narcissism is a relatively stable individual trait, mainly demonstrated as grandiosity, egoism, and self-inflation (Campbell et al., 2006). It is generally accepted that narcissism is a heterogeneous structure composed of grandiose and vulnerable (Wink, 1991; Morf and Rhodewalt, 2001; Miller et al., 2011). Both of them contain several common characteristics, such as self-centeredness and exaggerated self-importance. Vulnerable narcissists are low extroverted (Maciantowicz and Zajenkowski, 2018), and they are described as defensive, highly sensitive, and high shame proneness (Wink, 1991). Most research on narcissism has focused on grandiose narcissism (Gentile et al., 2013; Watts et al., 2013; O’Reilly and Hall, 2021; Hart et al., 2022), which is characterized by high self-esteem and self-confidence (Ksinan et al., 2021). Such kind of narcissism is associated with higher extroversion (Miller et al., 2011) and manifests through exploitative and aggressive behavior (Pincus et al., 2009). According to social cognitive theory, individuals’ cognitions of external events are influenced by individual characteristics (Bandura, 1986). Douglas et al.’s (2008) research also showed that the level of cognitive elaboration varies with the nature of the triggering event as well as individual differences. Thus, given that grandiose narcissistic individuals tend to overestimate their own abilities (Wink, 1991; Pincus et al., 2009; Miller et al., 2011), we propose that grandiose narcissism strengthens the positive relationship between i-deals and hubristic pride.

First, current studies have pointed out that some common characteristics of narcissism include fantasies about power (Joubert, 1998), superiority, and privilege (Miller and Josephs, 2009). Since the successful negotiation of i-deals may trigger the receivers’ sense of privilege and superiority (Xia et al., 2021), receiving i-deals could reinforce highly grandiose narcissistic employees’ sense of privilege and superiority. Thus, they may experience a higher level of hubristic pride than low grandiose narcissists who are less enthusiastic about privilege and superiority. Second, studies show that grandiose narcissists are inclined to overestimate their own abilities (Wink, 1991; Miller et al., 2011; Zajenkowski et al., 2018), and tend to interpret the success of events as a result of their own abilities (Tracy and Robins, 2007). Thus, employees low in grandiose narcissism may evaluate their abilities more objectively, and view i-deals as a joint result of effort and ability. Compare to highly grandiose narcissistic employees, those employees may experience lower level of hubristic pride when they receive i-deals. Finally, existing research has indicated that individuals with high grandiose narcissism tend to seek out opportunities to gain attention and admiration, as well as to maintain an inflated self-assessment (Ksinan et al., 2021). Given that success in negotiating i-deals with employers means that the i-dealers can enjoy more competitive and limited organizational resources than others (Liao et al., 2016), grandiose narcissistic i-dealers may experience inflated self-cognition, such as high level of hubristic pride. Thus, we propose the following hypothesis:

H4. Grandiose narcissism moderates the effect of i-deals on hubristic pride, such that this effect is stronger for employees with higher grandiose narcissism.

Furthermore, we argue that grandiose narcissism can moderate the indirect effects of i-deals on employees’ unethical behavior via hubristic pride. As mentioned before, social cognitive theory points out that the environment affects individuals’ cognition and behavior, and these effects vary with personality traits (Bandura, 1986). According to the arguments of social cognitive theory and hypotheses 1–4, highly grandiose narcissistic employees are likely to experience more hubristic pride after obtaining i-deals, and hubristic pride may weaken the self-moral restraint on employees, leading to more unethical behavior. The operation of this whole mechanism is self-organized, in which grandiose narcissism is the boundary condition for i-deals to produce negative effects, and hubristic pride, triggered by the cognition of i-deals, is the intermediary bridge that drives employees’ unethical behavior. Hence, we propose the following hypothesis:

H5. Grandiose narcissism moderates the indirect effect of i-deals on employees’ unethical behavior via hubristic pride, such that this indirect effect will be stronger for employees with higher grandiose narcissism.

## Materials and methods

### Sample and procedure

Before the formal investigation, we got ethical approval from the Ethical Committee of Business School, Qingdao University, and we conducted the investigation based on the guiding principles of the Declaration of Helsinki. Our data came from the employees in key positions in a large enterprise in Shandong Province, China. The employees of this company are at high risk of contracting the COVID-19 virus and face a lot of physical and psychological stress. We have taken strict protective measures throughout the investigation to ensure the safety of the investigators and participants. The survey was conducted in September of 2020. At the beginning of the investigation, in order to obtain the approval of the enterprise’s CEO, we first explained to him that the investigation will not disrupt the normal operation of the organization. Besides, we promised that the survey data would only be used for academic research and ensured the confidentiality of the results. Then, we obtained a list of participants from the human resources department and prepared an envelope containing the questionnaire and respondent instructions. It is worth noting that our questionnaires contained both forward and reverse order, which were randomly loaded into the envelopes issued to the respondents to balance the order effect of the items.

In the formal survey, we invited respondents to a large conference room. To reduce respondents’ guesses about the survey, we clarified that the survey results were used for academic research and would not be shared with organizations. In addition, to ease their concerns about the questionnaire and protect the privacy of the respondents, we emphasized to the participants that the questionnaire was completely anonymous. After completing the questionnaire, they sealed it in that envelope and handed it to the investigators. A total of 557 paper questionnaires were sent out and 492 valid questionnaires were completed, with a response rate of 88.33%.

### Measures

Strictly following the back-translation method proposed by Brislin (1980), we translated all the English-version scales into Chinese-version scales. All items were measured on a 7-point Likert-type scale, with 1 (strongly disagree) to 7 (strongly agree).

#### Idiosyncratic deals

I-deals were assessed by the 6-item scale developed by Hornung et al. (2008). The sample items are “I have received special training opportunities that are different from my colleagues” and “I have received individually customized work schedule that are different from my colleagues.” In this study, the Cronbach’s α of this scale was 0.93.

#### Grandiose narcissism

Grandiose narcissism was assessed by the 16-item scale developed by Ames et al. (2006). A sample item is “I know I am good because everyone says so.” In this study, the Cronbach’s α of this scale was 0.98.

#### Hubristic pride

Hubristic pride was assessed by a 7-item scale developed by Tracy and Robins (2007). A sample item is “I think I’m a little cocky.” In this study, the Cronbach’s α of this scale was 0.90.

#### Unethical behavior

Unethical behavior was assessed by a 5-item scale developed by Paterson and Huang (2019). A sample item is “I use excessive personal time, such as lunchtime, breaking time from work, or leaving the company for personal reasons.” In this study, the Cronbach’s α of this scale was 0.87.

#### Control variables

Prior research on i-deals has shown that demographic variables such as age, gender, organizational tenure, and education level of focal employees should be controlled when exploring the process of i-deals affecting focal employee behaviors (Liu et al., 2013). Therefore, following previous studies (Hornung et al., 2010), we selected these four demographic variables as control variables in our study.

### Demographics details

Among the valid samples, 49.59% were female and 50.41% were male. Their average age was 38.08 years old, with the most respondents aged 31–40 years, followed by 41–50 years. In terms of education, 46.95% held an undergraduate degree and 13.41% held a postgraduate or above degree. The responses showed that the average organizational tenure of the surveyed employees is 8.58 years (*SD* = 6.71), with organizational tenures ranging from 1–5 years (41.87%) and 6–10 years (35.57%). The details are given in Table 1.

**TABLE 1:** Demographics analysis (*N* = 492).

Demographics	Frequency	Percentage
**Gender**		
Female	244	49.59%
Male	248	50.41%
**Age**		
18–25	20	4.07%
26–30	94	19.10%
31–40	191	38.82%
41–50	136	27.64%
51 and above	51	10.37%
**Education**		
Technical secondary school and below	48	9.76%
Junior college	147	29.88%
Undergraduate	231	46.95%
Postgraduate or above	66	13.41%
**Organizational Tenure (years)**		
1–5	206	41.87%
6–10	175	35.57%
11–15	61	12.40%
>16	50	10.16%

## Results

### Discriminant and convergent validity

We used Mplus 7.4 to conduct confirmatory factor analysis to examine the discriminant validity of those four main variables in the conceptual model, including i-deals, grandiose narcissism, hubristic pride, and unethical behavior. As shown in Table 2, compared with one-factor model, two-factor model, and three-factor model, the proposed four-factor model showed the best fit indices (χ^2^ = 684.35, df = 521, CFI = 0.99, TLI = 0.99, RMSEA = 0.03, SRMR = 0.03), which met the critical values proposed by Hu and Bentler (1999).

**TABLE 2:** Results of confirmatory factor analysis (*N* = 492).

Model	χ^2^	df	χ^2^/df	CFI	TLI	RMSEA	SRMR
Four-factor model: ID; GN; HP; UB	684.35	521	1.31	0.99	0.99	0.03	0.03
Three-factor model 1: ID; GN; HP + UB	1075.50	524	2.05	0.96	0.96	0.05	0.04
Three-factor model 2: ID; GN + HP; UB	2643.04	524	5.04	0.85	0.84	0.09	0.18
Three-factor model 3: ID + GN; HP; UB	3083.23	524	5.88	0.82	0.81	0.10	0.18
Two-factor model 1: ID + GN + HP; UB	4946.98	526	9.41	0.69	0.67	0.13	0.22
Two-factor model 2: ID; GN + HP + UB	3924.45	526	7.46	0.76	0.74	0.12	0.21
One-factor model: ID + HP + GN + UB	5983.30	527	11.35	0.61	0.59	0.15	0.23

ID, idiosyncratic deals; GN, grandiose narcissism; HP, hubristic pride; UB, unethical Behavior; “+” represents the combination of factors; CFI, comparative fit index; TLI, Tucker–Lewis index; RMSEA, root mean square error of approximation; SRMR, standardized root means square residual.

We tested the results for factor loadings, AVE, Composite and Cronbach α reliabilities using SPSS 22.0. As shown in Table 3, the factor loadings of all items were higher than 0.60 (Bagozzi, 1981), and the reliabilities were higher than 0.70. The values of AVE were above 0.50 (Fornell and Larcker, 1981). All the values in this study were above the threshold, which indicated good reliability and validity.

**TABLE 3:** Factor loadings, AVE and reliabilities (*N* = 492).

Variables	Factor	Loadings	Cronbach alpha	Composite reliability	AVE
Grandiose narcissism	GN1	0.89	0.98	0.97	0.76
	GN15	0.88			
	GN14	0.87			
	GN11	0.87			
	GN3	0.87			
	GN16	0.87			
	GN7	0.87			
	GN12	0.86			
	GN2	0.86			
	GN10	0.86			
	GN13	0.86			
	GN4	0.86			
	GN6	0.86			
	GN9	0.86			
	GN8	0.86			
	GN5	0.85			
Idiosyncratic deals	ID1	0.89	0.93	0.94	0.73
	ID3	0.86			
	ID5	0.86			
	ID6	0.85			
	ID2	0.85			
	ID4	0.83			
Hubristic pride	HP2	0.82	0.90	0.92	0.61
	HP1	0.79			
	HP7	0.79			
	HP5	0.78			
	HP6	0.78			
	HP3	0.76			
	HP4	0.75			
Unethical behavior	UB1	0.87	0.87	0.91	0.66
	UB5	0.83			
	UB3	0.81			
	UB4	0.78			
	UB2	0.76			

### Common method variance test

Since all the variables used in this study were self-reported, we conducted Harman’s single-factor test to examine the common method variance by using SPSS 22.0. The results showed that four common factors with characteristic values greater than 1 were identified. All the extracted factors accounted for 70.69% of the total variance, and 35.25% of the variance was accounted for by the first factor. Therefore, common method variance was not a serious problem in this study.

### Descriptive statistics

The means, standard deviations, and correlation coefficients among all variables in this study are shown in Table 4. I-deals were significantly positively correlated with hubristic pride (*r* = 0.64, *p* < 0.01), and hubristic pride was significantly positively correlated with unethical behavior (*r* = 0.65, *p* < 0.01). In addition, the square root of the average variance extracted (AVE) of each construct in this study was greater than the inter correlations between constructs in the proposed model, which further indicated a good discriminant validity.

**TABLE 4:** Descriptive statistics and correlation analysis (*N* = 492).

	*M*	*SD*	1	2	3	4	5	6	7	8
(1) Gender	1.50	0.50	–							
(2) Age	38.08	8.78	0.02	–						
(3) Education	2.38	0.87	0.06	−0.45**	–					
(4) Organizational tenure	8.58	6.71	0.07	0.36**	−0.11*	–				
(5) Idiosyncratic deals	3.61	0.98	0.00	0.07	−0.04	0.13**	(0.86)			
(6) Grandiose narcissism	3.87	1.40	−0.05	0.02	−0.11*	−0.03	0.09*	(0.87)		
(7) Hubristic pride	3.63	0.94	−0.02	0.11*	−0.09	0.10*	0.64**	0.12**	(0.78)	
(8) Unethical behavior	3.35	1.06	−0.05	0.12*	−0.11*	0.09	0.64**	0.23**	0.65**	(0.81)

The data in diagonal brackets is square root of the AVE. Gender: 1 = male, 2 = female; Education: 1 = technical secondary school and below, 2 = junior college, 3 = undergraduate, 4 = postgraduate and above.

*p < 0.05, **p < 0.01.

### Hypothesis testing

To test Hypotheses 1–2, we conducted a hierarchical regression analysis. Results were presented in Table 5. We found that the direct effects of i-deals on hubristic pride (β = 0.64, *p* < 0.001, Model 6) and of hubristic pride on unethical behavior (β = 0.64, *p* < 0.001, Model 3) were significant, supporting Hypotheses 1 and 2.

**TABLE 5:** Results of hierarchical regression analysis (*N* = 492).

Variable	Unethical behavior				Hubristic pride			
	Model 1	Model 2	Model 3	Model 4	Model 5	Model 6	Model 7	Model 8
Gender	−0.05	−0.03	−0.03	−0.03	−0.03	−0.02	−0.02	−0.01
Age	0.06	0.05	0.02	0.02	0.07	0.06	0.06	0.05
Education	−0.07	−0.06	−0.04	−0.04	−0.05	−0.03	−0.03	−0.02
Organizational tenure	0.06	0.07	0.01	0.02	0.08	0.00	0.00	0.01
Idiosyncratic deals		0.63***		0.38***		0.64***	0.63***	0.57***
Hubristic pride			0.64***	0.40***				
Grandiose narcissism							0.05	0.04
Idiosyncratic deals × grandiose narcissism								0.29***
*R* ^2^	0.02	0.14	0.42	0.43	0.02	0.42	0.42	0.50
Δ*R*^2^	0.02	0.12	0.40	0.29	0.02	0.40	0.00	0.08
*F*	2.81*	16.40***	71.17***	61.46***	2.44*	69.86***	58.78***	69.11***

*p < 0.05, ***p < 0.001.

According to the suggestions of Baron and Kenny (1986), the existence of mediation effect should meet the following conditions: First, the independent variable has a significant influence on the mediator; second, the independent variable has a significant influence on the dependent variable; third, the mediator has a significant influence on the dependent variable. After both independent variable and mediator are added into the regression equation, if the effect of the mediator on the dependent variable is significant and the effect of the independent variable becomes insignificant, it is a complete mediation. On the contrary, if the effect of the mediator is significant, the effect of the independent variable is significant but becomes weak, it is a partial mediation.

As shown in Table 5, we found that the direct effects of i-deals on both hubristic pride (β = 0.64, *p* < 0.001, Model 6) and unethical behavior (β = 0.63, *p* < 0.001, Model 2) were significant. Adding hubristic pride to the regression results of Model 2, hubristic pride had a significant positive effect on unethical behavior (β = 0.40, *p* < 0.001, Model 4), while the impact of i-deals on unethical behavior (β = 0.38, *p* < 0.001, Model 4) was weakened. Therefore, hubristic pride played a mediating role in the relationship between i-deals and unethical behavior, supporting Hypothesis 3.

**FIGURE 2:**
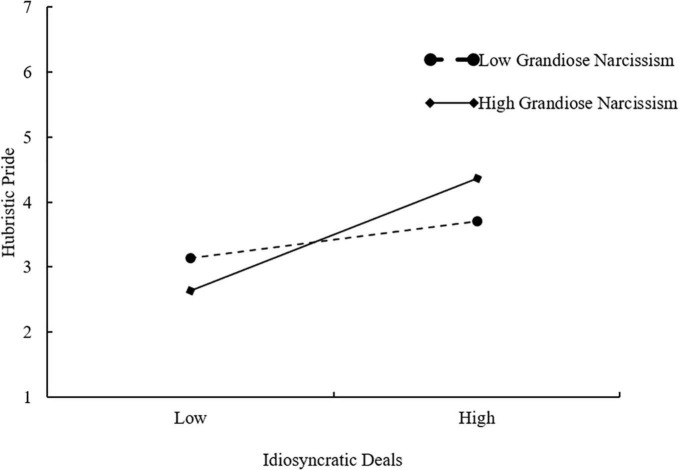
The interactive effect of idiosyncratic deals and employee grandiose narcissism on employee hubristic pride.

To test Hypothesis 4, we used the hierarchical regression method to examine the interactive effect of i-deals and grandiose narcissism hubristic pride. As shown in Table 5, the interaction term of i-deals and grandiose narcissism had a significant positive effect on hubristic pride (β = 0.29, *p* < 0.001, Model 8). Following the recommendation by Aiken and West (1991), we plotted simple slopes for values at 1 SD above and below the mean of grandiose narcissism. As shown in Figure 2, the positive effect of i-deals on hubristic pride is stronger for recipient employees with higher grandiose narcissism. Thus, Hypothesis 4 was supported.

Following the suggestion of Preacher et al. (2007), we used the Process program developed by Hayes (2013) with a 5,000-resample bootstrap method to test Hypothesis 5. Results reported in Table 6 showed that the effect was significant in high grandiose narcissism (β = 0.37, 95% CI = [0.29, 0.46], excluding 0), and in low grandiose narcissism (β = 0.12, 95% CI = [0.07, 0.17], excluding 0). The difference between the two indirect effects was also significant (effect = 0.25, 95% CI = [0.17, 0.34]), indicating that grandiose narcissism moderated the mediating effect of hubristic pride, supporting Hypothesis 5.

**TABLE 6:** Mediating effects and 95% confidence intervals at different levels of grandiose narcissism.

Moderating variables	Conditional indirect effects		
	Effect	*SE*	95% CI
High grandiose narcissism (+1 *SD*)	0.37	0.05	[0.29, 0.46]
Low grandiose narcissism (−1 *SD*)	0.12	0.03	[0.07, 0.17]
Difference	0.25	0.04	[0.17, 0.34]

## Discussion

Based on social cognitive theory, we advanced a moderated mediation model to explore the mechanism and boundary condition of i-deals on focal employee unethical behavior in the context of the COVID-19 pandemic. The results show that i-deals positively affect hubristic pride, which further positively predicted their unethical behavior in the organizations. Hubristic pride mediates the relationship between i-deals and unethical behavior. In addition, grandiose narcissism positively moderates the positive relationship between i-deals and hubristic pride, as well as the mediating role of hubristic pride between i-deals and employees’ unethical behavior.

### Theoretical implications

Our research made three theoretical contributions to the current literature. First, we explored and validated the potential negative impact that i-deals might have on the focal employees in the context of the COVID-19 pandemic. The great majority of previous studies suggest that i-deals play a positive role in enhancing focal employees’ affective commitment to the organization and improving their job satisfaction and job performance (Liu et al., 2013; Liao et al., 2016). However, we know surprisingly little about the potential negative effects of i-deals. Moreover, individuals’ psychological conditions and working behaviors changed a lot due to the COVID-19 pandemic (McFarland et al., 2020; Lin et al., 2021), which makes the effects of i-deals more complex. By investigating the dark side i-deals, our research provided a more comprehensive and balanced understanding of i-deals’ outcomes.

Second, we contributed to i-deals literature by constructing a moderated mediation model that outlines the underlying mechanism, boundary conditions, and explicates how and when i-deals’ negative impacts occur. This research found that i-deals can improve recipients’ hubristic pride, which in turn, increase their unethical behavior. Such process verifies the key propositions of social cognitive theory. That is, environmental events could affect individuals’ cognition, and such cognition shapes their behaviors (Bandura, 1986). In addition, our research indicated that grandiose narcissism strengthens the effects of i-deals on its downstream. Such findings support social cognitive theory, which suggests that the effects of the external environment on cognition and behavior vary among different individuals (Bandura, 1986). By examining the mediating role of hubristic pride and the moderating role of grandiose narcissism, this research responded to Liao et al.’s (2016) call to investigate more mechanisms through which i-deals affect potential outcomes.

Finally, we contributed to i-deals literature by investing the influence of i-deals on unethical behavior from the perspective of social cognitive theory. Most of the existing research that explores the impact of i-deals on the recipients are mainly based on social exchange theory (Ng and Feldman, 2015; Singh and Vidyarthi, 2018; Probst et al., 2021), social comparison theory (Marescaux et al., 2019a; Kong et al., 2020; Zhang et al., 2021), and self-enhancement theory (Liu et al., 2013; Katou et al., 2020; Xu et al., 2021). Although these theoretical perspectives are suitable in explaining the relationship between i-deals and focal employees’ responses to a certain extent, they overlooked the “environment-cognitive-behavior” path. Therefore, based on social cognitive theory, we explored the mechanism and boundary conditions of the i-deals’ negative impact. Our research responded to Liao et al.’s (2016) call that applying new theoretical perspectives to further enhance i-deals’ research.

### Practical implications

From the practical perspective, our research revealed the psychological changes and behavioral responses of focal employees after receiving i-deals, which have several practical implications for organizations. First, in order to cope with the challenges posed by the COVID-19 pandemic, managers have negotiated more i-deals with key employees to meet their needs. However, in this study, we found the issue of i-deals that managers may ignore. That is, i-deals may trigger recipients’ negative psychological reactions and behaviors, which run, counter to the managers’ original purpose of authorizing i-deals. Therefore, in order to maximize the positive effects of i-deals, managers should deepen their understanding of i-deals in combination with the current complex environment affected by the COVID-19 pandemic, and comprehensively consider the content and implementation costs of i-deals as well as the possible negative impacts.

Second, this study found that focal employees’ cognition of i-deals would be affected by individual traits. Especially, focal employees with a higher level of grandiose narcissism are more likely to perceive the successful negotiation of i-deals as a result of their ability, and generate hubristic pride, which in turn elicits their unethical behavior that is detrimental to the organizations and other employees. Therefore, managers should be cautious when negotiating i-deals with employees, and fully consider the personalities and individual characteristics of employees. In addition, according to the findings of this research, we believed that managers should pay more attention to the level of employees’ grandiose narcissism, guiding them to make accurate self-evaluations. In doing so, it can reduce grandiose narcissistic employees’ high expectations for special treatment, make them view i-deals with an objective attitude, and then reward the organizations with better work performance.

Third, existing studies found that in the context of the COVID-19 pandemic, most employees have experienced varying degrees of anxiety (Trougakos et al., 2020) and job insecurity (Latorre et al., 2021). These negative psychological conditions such as anxiety and depression are often associated with hubristic pride (Tracy et al., 2010), which will adversely affect the employees, the organizations as well as other team members. Therefore, during the COVID-19 pandemic, managers not only should pay attention to the performance of employees but also to their psychological conditions. In order to avoid employees’ negative psychological and behavioral reactions, managers could use some effective emotional coping methods, such as training and intervention programs to meet employees’ psychological needs, as well as providing emotional and instrumental support for employees, to help them better cope with the challenges caused by the COVID-19 pandemic.

### Limitations and future research

Our study may have several potential limitations. First, we used self-reported variable measures to collect data, which may cause common method bias (Podsakoff et al., 2003). Although the data analysis results showed that the problem of common method bias in this study was not serious, future research is encouraged to ask supervisors and coworkers to rate focal employees’ behaviors. And the experience sampling method can be used to track the psychological state and behavioral results of employees, so as to improve the reliability of research conclusions. In addition, the sample of this study came from an enterprise in Shandong Province, and the external validity of the research conclusions may be limited. Future research can be carried out in other regions affected by the outbreak.

Second, we adopted the cross-sectional data, which ignored the influence of time on the relationship between variables. At the beginning of the survey, we did not know how long the COVID-19 pandemic would last. In future studies, multi-point data collection can be used to further verify the causal relationship between variables. For example, future research can use the time interval method to obtain variable data in multiple batches, or the empirical sampling method to track the dynamic relationship among i-deals, hubristic pride, unethical behavior, and grandiose narcissism.

Third, in this study, we investigated i-deals recipients’ cognitive processes for their i-deals. Future research can explore the mechanism through which i-deals negative affect employees’ psychology and behavior from other theoretical perspectives such as self-validation theory. In addition, since we only explored the moderating role of grandiose narcissism in the negative impact of i-deals, the impact of i-deals on recipients can be explored from other personality traits in the future, such as two other Dark Triad (Paulhus and Williams, 2002), Machiavellianism (Christie and Geis, 1970), and psychopathy (Hare, 1991).

## Conclusion

The COVID-19 pandemic has brought enormous challenges to the human resource management of organizations, and thus the organizations hope to cope with these challenges by negotiating i-deals with employees. Based on social cognitive theory, we developed a model to explore the mechanism and boundary conditions of the potential negative effect of i-deals on the focal employees in the context of the COVID-19 pandemic. Our findings reveal that employees with high grandiose narcissism tend to experience more hubristic pride after obtaining i-deals during the COVID-19 pandemic, which in turn increases their unethical behavior. Therefore, we hope that our research can bring some new inspirations to scholars and managers. Specifically, the organizations should authorize i-deals with caution and use i-deals flexibly according to employees’ personality traits, so as to maximize its positive effects of i-deals and minimize the negative effects. We also invite future studies to conduct additional investigations based on this study, such as collecting data in different regions to verify the conclusion of this paper or analyzing whether other personality traits may affect individuals’ cognition of i-deals.

## Data availability statement

The raw data supporting the conclusions of this article will be made available by the authors, without undue reservation, to any qualified researcher.

## Ethics statement

The studies involving human participants were reviewed and approved by Ethical Committee of Business School, Qingdao University. The participants provided their written informed consent to participate in this study.

## Author contributions

ZL contributed to the preparation of the manuscript. XZ contributed to the design of the research model. HX and HD contributed to the analysis and interpretation of the data. JL contributed to the collection of the data. YL contributed to the design of the research model and the revision of the manuscript. All authors contributed to the article and approved the submitted version.
